# Digital Informal Care: The Use of Technology in Family Care. A Scoping Review

**DOI:** 10.3389/phrs.2025.1608872

**Published:** 2026-01-23

**Authors:** Vasileios Nittas, Alexia Bikou, Jana Sedlakova, Andreas Hellmann, Viktor Von Wyl, Milo Alan Puhan

**Affiliations:** 1 Epidemiology, Biostatistics and Prevention Institute, University of Zurich, Zurich, Switzerland; 2 Department of Informatics, University of Zurich, Zurich, Switzerland; 3 Entyre GmbH, Berlin, Germany; 4 Institute for Implementation Science in Health Care, University of Zurich, Zurich, Switzerland

**Keywords:** informal care, digital health, mobile health, home care, family caregiving

## Abstract

**Objectives:**

To map the recent literature on digital health in informal caregiving, identify commonly used technologies, their functions and impact, as well as barriers and facilitators.

**Methods:**

We searched Medline, Web of Science, and CINAHL for randomized controlled trials and quasi-experimental, observational, and qualitative studies, published in English between 2019 and 2024.

**Results:**

110 studies were included, most of which targeted informal caregivers in dementia care and used moderately complex, consumer-facing technologies for education and caregiving support. Positive impact was reported on various outcomes such as caregiver burden, psychological wellbeing, caregiver competence, quality of life, caregiver-patient relationships, as well as care coordination and efficiency. Barriers included limited digital literacy, technical issues, low accessibility, caregiving burden, and data security concerns. Facilitators were good digital skills, social and emotional support, user-friendly designs, and perceived usefulness.

**Conclusion:**

Digital informal care is emerging and shows promise in supporting informal caregivers by improving their wellbeing, skills, and connectedness. However, barriers and knowledge gaps remain, highlighting the need for additional research as well as more inclusive and person-centred digital informal care approaches.

## Introduction

As global demographics shift, the number of people with multiple chronic conditions and in need of long-term care (LTC) will continue rising, as will the demand for informal care [1]. Informal care is defined as the provision of care for a relative, friend, or acquaintance without an official contractual agreement [2]. In Europe, it forms the cornerstone of care systems, contributing to as much as 80% of all delivered LTC [3]. With the ever-growing shortages of trained care staff, informal caregivers provide an invaluable public service, reducing the burden on health systems, preventing early institutionalization, and keeping patients longer at home [4]. Yet, they often carry a significant financial, physical, and mental health burden [5]. Previous research indicates that providing informal care leads to a higher risk for anxiety, depression, social isolation, and an impaired quality of life [6–8]. Reducing some of that risk requires targeted support and resources [7, 9, 10]. Thus finding innovative ways to strenghten informal care and support both informal caregivers and LTC patients is crucial.

Digital health, defined as the use of information and communication technologies for health-related purposes, has the potential to support informal caregiving by providing tools that enhance care efficiency and quality, reduce burden, and improve outcomes [11]. For example, personalized smartphone applications (apps) can support informal caregivers with symptom tracking, fast communication with healthcare professionals, as well as personalized care plans, reminders, and other support functions [12, 13]. Previous research shows that digital and mobile tools can be a cost-effective and convenient way to help caregivers build communities, receive support, and access resources [14]. Common functions include (a) patient health summaries, (b) educational information (including decision support), (c) resources and services for caregivers, (d) solutions to common problems during care (problem-solving support), (e) questionnaires to assess caregivers’ wellbeing, and (f) data collection and monitoring [12, 15]. Previous systematic reviews have also shown that digital health tools have the potential to improve caregiver outcomes (e.g., depressive symptoms, anxiety, self-efficacy, caregiving skills, quality of life, social support, and problem-coping abilities) but highlight the need for more research, particularly with longer follow-up periods and with a focus on how specific digital components (e.g., personalization) impact informal care [11, 16–19].

This scoping review aims to map the recent scientific literature on the use of digital health in informal caregiving, hereafter also referred to as digital (informal) care. More specific objectives are: [1] to map the technologies used and their functions [2], to summarize their different roles in informal care, and [3] to identify related barriers and facilitators.

## Methods

We conducted a scoping review of published primary research. Our methodology was systematic and was guided by Arksey and O’Malley’s framework, as well as Levac, Colquhoun, and O’Brien’s conceptual extensions [20, 21]. A protocol was registered prospectively [22].

### Search Strategy, Selection Criteria, and Screening

A purposely sensitive search strategy was designed and tailored to three electronic databases: Medline, Web of Science, and CINAHL. Searches were run in January 2025 to include publications until December 2024. We used the following term variations of informal care and digital health: family care, informal care, home care, parental care, spousal care, technology, digital, mobile health, smartphone, wearable, smartwatch, e-health, internet, web-based, computer-based, online, chatbot, virtual, app, telemedicine, and artificial intelligence. Our database-adapted search strategies are provided in Supplementary Material S1.

We included randomized controlled trials (RCTs) and studies with quasi-experimental, observational, and qualitative study designs, published in English between 2019 and 2024. Additionally, eligible studies must address informal care and include at least one patient or caregiver outcome. Screening was conducted in duplicate across two stages. Three independent reviewers (VN, JS, AB) first screened titles and abstracts and then full texts in duplicate. Studies not fulfilling all the above selection criteria were excluded. Any disagreements were resolved by a third reviewer (AH). All screening stages were conducted in the web- and mobile-based platform, Rayyan [23].

### Data Extraction

Data extraction was conducted using Elicit (accessed March–June 2025) [24]. All extraction items were pre-defined by the study team. For each item, a detailed prompt was designed and entered into Elicit’s automatic data extraction tool. Prompts were carefully designed to generate high-accuracy extractions, avoid redundancies, and maintain consistency. For the first ten studies, data extraction was conducted with Elicit and also manually, in duplicate by two reviewers (VN, AB), ensuring that the prompts led to accurate and consistent data extractions. Prompts were then corrected and adjusted, and the process was repeated with one reviewer (VN) until satisfactory accuracy was reached for all data items. Extractions were quality-checked manually by one reviewer (AB). All data extraction items and their corresponding Elicit prompts are provided in Supplementary Material 2.

A key data extraction item was technology complexity, divided into function and interaction complexity. The function complexity of technologies was assessed based on the number of functions/features, their interlinkage, and the degree of personalization. Technologies were rated as simple, if they contained of a single function with minimal features (e.g., step counters, basic symptom trackers); as moderate, if they contained multiple, interlinked features but without personalization (e.g., apps combining fitness tracking with dietary recommendations), and complex if they consisted of larger systems with advanced features such as AI-driven decision support, interoperability with other platforms, or dynamic personalization (e.g., telemedicine platforms integrated with electronic health records).

The interaction complexity of technologies was rated based on the required interaction intensity between users and technologies as well as the mode of data collection. Technologies were rated as simple if they required minimal user interaction and primarily passive data collection (e.g., wearable devices that automatically collect data); as moderate if they required more frequent yet irregular interaction, such as periodic active data input (not daily) or responding to prompts (e.g., apps requiring user-provided symptom logs); and as complex if they required frequent interaction, active data collection, or if users needed training for effective use, possibly involving multiple stakeholders (e.g., platforms for remote monitoring, with manual data input and customizable dashboards).

### Data Synthesis and Reporting

We synthesized our findings following a qualitative and iterative thematic approach, conducted by one reviewer and quality-checked by a second reviewer [21]. After data familiarization, we generated initial data-derived themes. Emergent themes were then manually summarized and clustered, generating conceptual maps that provided an overall picture of our findings. Outlier findings (e.g., topics mentioned only a single or a few times) or topics not fitting into one of the overall themes were considered equally significant and synthesized separately. Reporting was guided by the Preferred Reporting Items Extension for Scoping Reviews (PRISMA-ScR) statement [25].

## Results

**FIGURE 1:**
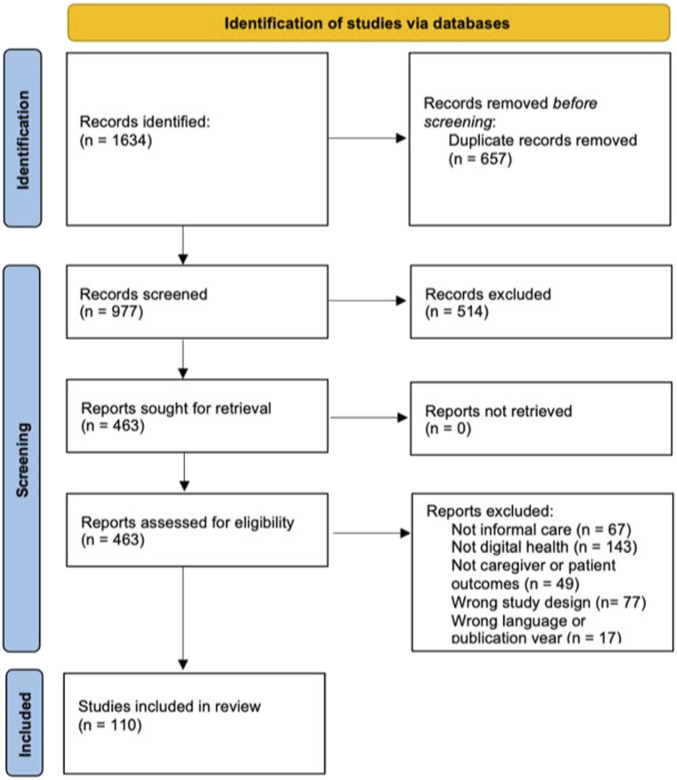
PRISMA 2020 flow diagram (Zurich, Switzerland.2025).

We identified 1,634 articles and screened the titles and abstracts of 977 studies. We excluded 514 studies at the title and abstract stage and retrieved 463 full texts. Finally, 110 studies addressing digital informal care were deemed eligible and were included (see Figure 1, PRISMA 2020 Flow Diagram) [26]. All included studies are provided in Supplementary File 3.

Between 2019 and 2024, research on digital informal care has gradually increased, with nine publications in 2019, 14 in 2020 and 2021, 24 in 2022, 28 in 2023, and 21 in 2024. Most (n = 50) included studies were conducted in Europe, among which Sweden (n = 9) and the Netherlands (n = 8) had the highest number of publications. North American studies, conducted in the U.S. (n = 27) and Canada (n = 9), were the second most common geographic areas, followed by Asian studies (n = 18), among which Iran (n = 5) and China (n = 4) were most frequent. Finally, seven studies were conducted in Australia. Most included studies had either qualitative (n = 30) or experimental designs (n = 27), followed by quasi-experimental (n = 24), mixed-methods (n = 22), and observational (n = 7) designs.

### Participant and Study Characteristics

Most (n = 86) identified research targeted informal caregivers. The remaining studies (n = 24) included caregivers and their patients, of which only five were RCTs. In terms of care context, most (n = 48) studies addressed dementia care, followed by chronic disease (n = 43), mental health (n = 10), and acute disease/injury (n = 6) care. The caregiver-patient relationship was reported in 82 studies. Informal caregivers were most commonly either first-degree relatives or spouses (n = 74) providing home-based care (n = 95), with only eight studies additionally including second- and third-degree relatives or friends. Most research focused primarily on middle-aged (n = 54) and female (n = 68) caregivers, followed by older age groups (n = 28) and mixed-gender samples (n = 29). Most digital informal care research lasted several weeks to a few months (median 8 weeks), with an overall substantial variation across studies. Most research required a daily or weekly use of technology, again with a wide variability and a mean of about 3 interactions (e.g., data input) per week. The socio-economic status (SES) of caregivers was reported in three studies, two of which focused on middle-income and one on low-income participants. Nationalities or ethnicities were reported in 43 studies, with most studies (n = 32) including only or mostly white participants. The employment status of informal caregivers was reported in 40 studies, with 24 studies including participants with and without employment, 15 studies including primarily employed and 5 studies primarily including unemployed participants. Common reasons for participant exclusion were psychiatric illness or cognitive impairments (n = 27), no access to or willingness to use technology (n = 26), lack of knowledge skills (n = 22), and certain physical deficits, such as visual or hearing impairments (n = 22).

### Aims of Digital Informal Care

The primary objectives of digital care technologies, as identified across the 110 studies, were categorized into ten key areas. Most (n = 73) technologies aimed to provide caregiver education as well as practical care support, such as reminders and support with daily care tasks. Many (n = 63) technologies also aimed to improve the psychological wellbeing of caregivers, often through interventions that provide emotional support or coping strategies, such as cognitive behavioural therapy and mindfulness training. This was followed by technologies that targeted caregiver burden (n = 42), self-efficacy (n = 37), general and health-related quality of life (n = 35), and socialization (n = 34). Other aims included fostering general wellbeing (n = 28) and the caregiver-patient relationship (n = 28), improving care coordination and communication with healthcare professionals (n = 24), as well as facilitating health monitoring and prevention (n = 21).

### Technology Features

Most (n = 61) studies included at least one web-based technology (e.g., app or software) such as online diaries, online coaching and health education platforms, personal health records, and chatbots. Mobile applications, primarily for smartphones and tablets, were included in 50 studies. Other technologies were smartwatches, wearables, sensors, virtual reality gear, and smart devices (e.g., TV, scale). Almost all (n = 109) studies included consumer-facing technologies, and most (n = 101) of them required an internet connection. The function complexity of most (n = 97) digital informal care was classified as moderate, incorporating multiple interrelated features, such as educational content, communication tools, and health monitoring capabilities without advanced automation. For instance, mobile applications often combined symptom management resources with social support features. Nine identified technologies were classified as having high function complexity, for example, integrating AI-driven decision support systems or real-time health analytics, which required more intensive user interaction and training. Only two technologies were classified as simple, relying on single functions, passive monitoring, or basic informational tools that require minimal user engagement. The interaction complexity of most technologies (n = 84) was also moderate (e.g., regular data input or engagement with educational modules), followed by complex (n = 20), including technologies with high interaction levels, particularly in systems involving multiple stakeholders like healthcare providers and family members.

Only 39 studies reported that informal caregivers, patients, and/or other stakeholders (e.g., health professionals) were actively involved in the design and development of digital informal care. Many papers referenced the use of co-design principles and methods, including interviews, focus groups, discussion sessions, surveys, regular feedback loops, and advisory panels. The provision of training and support was reported in more than half of our sample (n = 59), including onboarding instructions in the form of manuals and user guides, continuous monitoring and technical support (e.g., within apps, via telephone, and E-Mail), and structured training offerings (e.g., online tutorials, educational videos, and technology-literacy workshops). Finally, patient-reported outcomes were only addressed in 18 studies, primarily focusing on psychological outcomes (e.g., perceived stress, anxiety, depression), physical burden, and quality of life.

### Reported Outcomes

Experimental, quasi-experimental, and observational studies (n = 80) reported a) psychological and emotional outcomes, (b) functional and health-related outcomes, and (c) social and relational outcomes. Psychological outcomes primarily included reduced caregiver burden, reported in 16 (20%) studies, and improved psychological wellbeing (e.g., reduced depression, anxiety, and stress), reported in 29 (36%) studies. Better functional and health-related outcomes, such as improved caregiver competence, general and health-related quality of life, and physical health, were reported in 19 (24%), 13 (16%), and six (7.5%) studies, respectively. Most common social and relational outcomes were improved caregiver-care recipient dyad, strengthened social connectivity, and enhanced care coordination and efficiency, reported in 10 (12.5%), eight (10%), and four (5%) studies.

Some experimental, quasi-experimental, and observational studies reported no positive outcomes with various explanations. For some technologies, such as personal health records, web-based caregiver information resources, and online community platforms, low digital literacy and perceived usefulness/added value were the main reported barriers. For purely educational apps, the lack of interactivity was described as a potential reason for their low impact on caregiving outcomes. For web- and smartphone-based apps, primarily targeting dementia care, technical challenges and app complexity hindered their use, while very novel technologies (e.g., humanoid robots) are not familiar at all to caregivers and patients, also hindering uptake and engagement.

Qualitative studies (n = 30) highlighted that caregivers and patients appreciated technologies that were co-designed, user-centered, and tailored to meet individual needs (n = 11, 37%), as well as simple, user-friendly, and culturally appropriate (n = 6, 20%). Four studies (13%) reported improved caregiver-care recipient relationships, and four (13%) studies reported that technologies such as telepresence robots and social interactive features led to increased feelings of connectedness and socialization. Furthermore, caregivers described that digital care technologies allow for communication flexibility (e.g., through chats), help with self-reflection and self-monitoring, increase knowledge and self-efficacy, improve care coordination (e.g., through connecting with healthcare providers), and reduce negative emotions.

### Barriers and Facilitators

All studies mentioned at least one barrier or facilitator. Five core barriers were identified, outlined in detail in Table 1. Low digital literacy, defined by the European Information Society as “the awareness, attitude and ability of individuals to appropriately use digital tools and facilities (…),” and limited technology familiarity, referring to prior experience with or exposure to technology, were commonly mentioned barriers to technology acceptance and engagement [27, 28]. Moreover, technical and usability issues, that is, challenges in interaction with technology, such as malfunctions (e.g., bugs), poor navigation, and lack of personalization (e.g., missing adaptation to individual needs and preferences) were described as hindering continuous use. Low accessibility and inclusion, for example, because of language limitations or limited access to technology by certain subgroups (e.g., older caregivers, ethnic minorities), in combination with the limited capacities of caregivers to learn and use new technologies in parallel to their physically and emotionally straining care duties. Finally, concerns about data security and privacy, such as the protection of sensitive health data, were key ethical barriers. That highlights the seriousness of consequences resulting from data misuse, such as unintended disclosure of sensitive health information and discriminatory practices. Further ethical barriers were the lack of culturally sensitive technologies tailored to the cultural norms and values of users, as well as the lack of trust.

**TABLE 1:** Barriers to digital informal care (Zurich, Switzerland. 2025).

Barrier(s)	Examples
Digital skills and literacy	Low digital knowledge, low confidence in using technology, lack of experience with technology, lack of training and support, preference for non-digital methods
Technical and usability issues	Technical bugs, slow internet, device incompatibility, poor navigation, too many notifications, lack of personalization, lack of interactivity, high data entry effort
Accessibility and inclusion	Lack of language options, complex language, limited technology access, lack of adjustments to cognitive or physical limitations (e.g., non-adjustable font sizes)
Caregiving burden	Little time or energy to use/learn technology, difficult integration into daily routine, especially if learning curves and time commitments required
Privacy and trust (ethical issues)	Data security concerns, fear of losing human interaction, lack of cultural adaptations

Four key facilitators have been identified that enhance digital care technology acceptance and adoption, outlined in more detail in Table 2. Digital literacy and support (technological and social) by professionals and peers were reported as important factors for the successful integration of technologies in informal caregiving and for fostering a sense of belonging and reducing feelings of isolation among caregivers. Moreover, user-friendly technologies and designs were described as critical for encouraging engagement among caregivers and patients with varying levels of technological proficiency. High perceived technology value (e.g., for reducing care burden) along with accessible and inclusive design further enhances adoption and engagement.

**TABLE 2:** Facilitators of digital informal care (Zurich, Switzerland.2025).

Facilitator(s)	Examples
Digital literacy and support	Training sessions, help hotlines, experience with technology, support by tech-savvy family members or friends, support through peer groups
User-friendliness and person-centeredness	Intuitive and simple designs, personalized settings, offline functionality (printable materials, offline modes), blending human and digital interactions
Perceived value	Perceived need and usefulness of technology (e.g., in reducing caregiver burden), perceived added value for daily care duties
Accessibility and inclusion	Mobile-friendly applications and integration with existing devices and habits, flexible access and engagement options

## Discussion

Our findings provide an up-to-date overview of digital informal care research. The field is emerging yet still primarily focused on dementia care and chronic disease management, and targeting psychological, functional, and social outcomes. The identified technologies primarily target middle-aged female caregivers who are first-degree relatives or spouses providing home-based care.

### Literacy at the Core of Digital Informal Care

Digital and health literacy emerged as core components of digital informal care. Health literacy was a frequently reported objective, while digital literacy was a major determinant of technology use and acceptance by informal caregivers and LTC patients. While most identified technologies targeted literacy through educational components, these were often integrated with interactive features such as self-reflection, self-monitoring, peer support, and skills training to foster self-efficacy and support with daily care tasks. This underscores a broader shift from mere information transfer toward digital interventions that foster concrete competencies and actions through proactive support measures. This is aligned with previous systematic reviews and meta-analyses reporting favorable effects for digital health interventions with integrated educational and interactive components such as feedback loops, monitoring tools, skills training, peer support, and healthcare provider communication [29, 30]. Overall, these findings suggest that the mere provision of medical information is often insufficient for informal caregivers and patients, who may benefit more from technologies with multiple interactive features targeting self-reflection and self-efficacy.

Simultaneously, most identified digital tools were classified as moderately complex, with multiple interconnected features that require regular (e.g., daily) user engagement (e.g., entering information into an app). However, only about one-third of studies reported the active involvement of caregivers, patients, or other stakeholders in the design and development process of these tools, which inherently contrasts with digital and health literacy focus of most studies. This increases the risk that technologies fail to address the actual needs of informal caregivers and their patients. Furthermore, a lack of participatory design and development can exacerbate existing inequities, leaving caregivers and patients with lower digital and health literacy out [31]. To ensure that digital care reaches, benefits and meets the needs of the most underserved and vulnerable informal carers and patients, it is essential to combine literacy with accessibility, inclusivity, and participatory designs [31].

### Who Is Excluded From Digital Informal Care Research?

Our findings highlight that several population groups might be underrepresented in the current digital informal care literature such as LTC patients and their informal caregivers with (a) no access to or willingness to use technology, (b) lack of digital knowledge and skills, and (c) certain physical deficits such as visual or hearing impairments. This reflects previous findings, which show that certain population subgroups, such as those with lower education, less income, older age, and any form of disability, are often excluded and tend to benefit less from the internet and technology [32, 33]. We found a reporting gap of socioeconomic data (only three studies reported socioeconomic status), making it difficult to assess whether vulnerable subgroups (e.g., low-income informal caregivers) receive the digital support they need.

Ethnic and cultural minorities are also underrepresented in recent digital informal care research. In many countries, such as the U.S., a significant proportion of informal care is provided within ethnic minority communities, who often provide family care while working and facing higher financial and structural hurdles [34]. Yet these are the groups that are most often excluded from digital health research, potentially widening existing disparities. Current evidence suggests that culturally sensitive technologies promote fairness, autonomy, respect for diverse values, and ensure the inclusion of marginalized populations. To support inclusivity and equality, value-sensitive and ethically informed design frameworks, as well as trauma-informed principles, could be applied to proactively promote reflection and attention to users’ needs and values [35–39]. This aligns with previous research that highlights the benefits of user-friendly and culturally sensitive designs [40, 41].

### Implications for Practice and Future Research

Our findings highlight that providing technical and social support and user-friendly designs that provide clear added value for caregivers and LTC patients are key to successful digital informal care. For those who develop digital care technologies, a stronger emphasis on co-design that considers culture and context, and includes currently underserved communities, is needed. Finally, digital care technologies may be better accepted and more impactful if they move beyond mere information provision to combine integration and interaction in ways that align with the complex needs and challenges of daily informal caregiving. Overall, our findings suggest that a multidimensional approach is required, combining technological development with appropriate support systems and education. In the context of a responder model, digital health technologies could serve as valuable tools for identifying risks early, for example, through monitoring systems that automatically identify risks in the context of certain care activities to help monitor and prevent complications and costly treatments.

Future research should prioritize co-designed digital informal care that actively involves caregivers and LTC patients in all phases of technology development. More attention should be devoted to developing tools that promote health literacy in interactive and engaging ways, rather than focusing on the mere provision of information. Such tools should empower caregivers to develop practical care skills, effortlessly monitor their own and their patients’ health, and better assess the care situation and needs of their patients. Longitudinal studies with longer follow-up periods are needed to evaluate the sustainability of digital informal care technologies, along with a stronger focus on making new technologies accessible and inclusive [11].

### Limitations of the Current Evidence Base

The current literature has several limitations. We limited our research from 2019 to 2024 which may have led to the exclusion of potentially valuable literature. Furthermore, most studies had small sample sizes, limiting statistical power and generalizability. The dominance of research from high-income countries, particularly from Europe and North America, further restricts generalizability. Future research should focus on low- and middle-income settings. The disease-specific focus, with 68% of studies addressing dementia or cancer care, leaves substantial gaps in our understanding of how digital technologies could support multimorbid LTC patients and their informal caregivers [42]. Most people receiving informal care are elderly, often living with multiple chronic conditions [42]. That requires a shift towards more research that evaluates digital informal care in the context of multimorbidity and not of a specific disease [42]. Finally, the outcomes assessed across studies were very heterogeneous, making comparisons difficult. The field would benefit from greater consensus on core priority outcomes to support comparability, evidence synthesis, and guide future implementation efforts.

### Conclusion

This scoping review highlights the emerging role of digital health in supporting informal caregivers and LTC patients, particularly in the context of dementia and chronic disease care. Most identified technologies were moderately complex, consumer-facing tools targeting health literacy, practical caregiving support, and psychological wellbeing, with evidence suggesting positive impacts across various outcomes such as caregiver burden, psychological wellbeing, caregiver competence, quality of life, caregiver-patient relationships, as well as care coordination and efficiency. However, our findings indicate that key barriers remain, including limited digital literacy, technical issues, accessibility concerns, and worries about data security, which disproportionately affect marginalized groups and those with lower digital skills. These barriers could be mitigated through a stronger focus on user-friendly and person-centred technologies that are culturally sensitive, interactive, and embedded within social and emotional support structures. Addressing current gaps and barriers is essential for facilitating a sustainable informal care ecosystem that benefits from digitalization.
