# Supplementary material for: Digital Informal Care: The Use of Technology in Family Care. A Scoping Review
Source: Public Health Rev. 2026 Jan 23;46:1608872. doi: 10.3389/phrs.2025.1608872 (PMC12876001; doi:10.3389/phrs.2025.1608872)
Supplement: Supplementary file 2 [file DataSheet2.docx]

**Supplementary File 2: Data extraction items and elicit prompts**

| Data extraction item | Elicit prompt |
| --- | --- |
| Year of publication / region | Give the year of study/paper publication. Not the year the study was conducted, but the year it was published online or in print. This number is not found in text but somewhere on papers corners.  Give the region of the study. If multiple countries or regions, list all of them. You can also say something like 'multinational' if there are multiple countries, but individual countries are not specified. Add "NA" instead of the country if the country is not specified.  Report as follows:  year/region |
| Methodology | Give the main study methodology (e.g., pretest-posttest randomised controlled trial) in a single bullet point. Do not extrapolate, and be as precise as possible. For example, do not just say "pilot study" but actually refer to the design, such as "pilot randomised controlled trial". |
| Study objectives | Describe the study objectives/goals/aims. Be concise and use bullet points if there are several goals to the study (numerate). Only mention objectives that are stated as such by the authors; don't extrapolate. Avoid redundancies and repetitions. |
| Participant count | Provide only the count of participants and add whether they fall under the category "patient" or "informal caregiver". If both are included, provide counts separately for patients and informal caregivers.  If randomization was done, give the number of participants randomized at the baseline or first phase of the study, not necessarily the number who were treated or analyzed. Do not give numbers across trial arms in case of RCT, just the total participant number.  First mention count and in brackets either (informal caregivers) or (patients). If both included report first count (informal caregivers) and below count (patients). |
| Patient-caregiver relation | Describe in 1-2 words the patient-caregiver relation. Provide only the following categories  first degree (for parents, full siblings, children)  second degree (for grandparents, grandchildren, aunts/uncles, nephews/nieces, half-siblings)  third degree (for first cousin, great-grandparent, great-aunt, great-uncle, great-niece, great-nephew, great-grandchild, half-aunt, or half-uncle)  Other/unspecified relative (for any relative that is beyond the third degree or the degree not clear from the manuscript)  spouse (for spouses and partners, married or unmarried)  friends (for friends, neighbors, and other acquaintances)  other/not specified (if none of above applicable or not clear from manuscript) |
| Disease profie/ care context | Disease profile: If the study has a focus on one or multiple specific disease(s)/condition(s)/or symptom(s) of patients and/or Informal caregivers please mention all (e.g., diabetes, chronic conditions, infectious diseases, HIV, high blood pressure etc.).Please always mention in brackets if disease is linked to patients or informal caregivers. Example: Late stage cancer (patients), burnout (informal caregivers)    Care-context: Here please provide any mentioned care context and always mention if home-based or hospital-based etc. (e.g. home-based palliative care, hospital based intensive care).  Report as show below:  1. Disease profile: you text  2. Care context: your text  Avoid redundancies. |
| Socio-demographic profile | Please describe the socio-demographic focus of the study and provide results im numbers only for categories shown below. If paper includes informal caregivers AND patients as participants, please report separately.  1. age: primarily younger (if paper explicitly mentions focus on younger adults or if at least 70% of participants is 40 years of age or below); primarily middle-aged (if paper explicitly mentions focus on middle-aged adults or if at least 70% of participants is between 41 and 65 years of age or below; primarily older (if if paper explicitly mentions focus on older adults or if 70% of participants is 66 years of age and above). Say "not reported" if not reported.  2. sex/gender: primarily females (if paper explicitly mentions focus on females or if at least 70% of participants female); primarily male (if paper explicitly mentions focus on males or if at least 70% of participants male); mixed (if paper does not describe any gender/sex focus and distribution rather equal). Say "not reported" if not reported.  3. nationality/ethnicity/race: If mentioned, provide a summary of ethnicities and races. Say "not reported" if not reported.  4. socio-economic status: here describe any broad socio-economic focus (e.g., low income, high-income)and only of specifically mentioned in the manuscript. Say "not reported" if not reported.  5. employment status: mostly employed (if at least 60% of participants employed); mostly unemployed (if at least 60% of participants unemployed), mixed (if both included), and not reported (if not reported)  6. education: simply list all levels of education of participants using the following levels: no education/illiterate, primary, secondary, tertiary. List all of levels of education and not only the most common. If only most common education provided then add this below "(most common)". Say "not reported" if not reported.  5. other: (anything not fitting in above categories, e.g., sexual orientation, religion, rural/urban residents, mostly living with patient, mostly living outside of patient home etc.)  (please report as shown above, no capital letters after numeration)  If the study includes patients and informal caregivers and the socio-demographic focus differs, describe separately for both groups. Do not mention anything about diseases or conditions here. No need to report separately for intervention and control groups (just report overall). If one of categories not reported please say "not reported".  Avoid redundancies and keep it simple. For example if 70% female say "mostly females". Provide means ages without further explanations. |
| Study exclusion | Mention all reported exclusion criteria for study participation (not exclusion criteria after study participation). Do not use acronyms. If study includes patients and informal caregivers, mention exclusion criteria separately. Numerate. Avoid redundancies. Do not use full sentences, just list exclusion criteria. If patients and informal caregivers included, list criteria separately for both groups (only if they differ). If only one group included, not need to mention that group, just lift exclusion criteria.  If none mentioned, just say "not reported" |
| Intervention duration | Please describe very briefly (a few words) the intervention duration (days, weeks, months etc.) and frequency of technology use (e.g., ad hoc, daily, weekly, monthly). If technology use frequency is contextual, for example (e.g., whenever stressed) then just describe that.  Report as follows  intervention duration / technology use frequency.  If if one of the two not specified, then replace with "NA". User numerical values for all numbers and do not write them out. |
| Technology | Provide the type of technology/technologies used in the study, including hard and software. Stay consistent, for example WhatsApp (smartphone app), bluetooth (software), Garmin (smartwatch/wearable). Numerate.  In a separate section describe whether any of the included technologies are consummer-facing ( refers to technological tools, products, or systems that are designed specifically for use by end consumers, rather than businesses or internal operations or research-grade devices).  In a separate section describe whether internet connectivity required or not.  Report as shown below:  1. ...  2. ...  3. ...  Consumer-facing technology included? Yes/No/Unclear  Internet connectivity required? Yes/No/Unclear  Please be concise and avoid redundancies. Do not mention anything twice. |
| Tech functionalities | Describe in simple numerated bullet sentences the main functionalities of the technology. For example  1. Educational modules for informal caregivers  2. Peer-chat for informal caregivers  3. Symptom-diary for patients  Be concise and avoid redundancies. |
| Technology complexity | Describe the level of technology complexity (overall for the study). Differentiate between functional complexity and interaction complexity.  For functional complexity:  (a) Simple: Single-function tools with minimal features (e.g., step counters, basic symptom trackers). Provide a 1-2 sentences explanation why in brackets.  (b) Moderate: Tools with multiple, interrelated features but limited automation or personalization (e.g., apps combining fitness tracking with diet suggestions). Provide a 1-2 sentences explanation why in brackets.  (c) Complex: Systems with advanced features like AI-driven decision support, interoperability with other platforms, or dynamic personalization (e.g., telemedicine platforms integrated with electronic health records). Provide a 1-2 sentences explanation why in brackets.  For interactional complexity:  Simple: Minimal user interaction required, often passive (e.g., wearable devices that automatically collect data). Provide a 1-2 sentences explanation why in brackets.  Moderate: Moderate interaction needed, such as periodic data input or responding to prompts (e.g., apps requiring user-provided symptom logs). Provide a 1-2 sentences explanation why in brackets.  Complex: High interaction or training required for effective use, possibly involving multiple stakeholders (e.g., healthcare professionals, patients, caregivers) (e.g., platforms for remote monitoring with customizable dashboards). Provide a 1-2 sentences explanation why in brackets. |
| User training/support provided? | Describe with a yes, no or not reported whether any type of technology use training or any other type of technology use support was provided to participants. If yes, give brief sentence on how training looked like. For "no" and "not reported" no need to add anything else. |
| Person-centered tech? | Does the paper mention or describe that any participants (patients and/or informal caregivers) were actively engaged in the development stages of the used technologies (e.g., participatory design was used to design the XY app were participants co-designed the features). If not mention, please say "not reported". |
| Tech barrers/facilitators | Describe any mentioned barriers regarding the use of technology. That can include any mentioned challenges, problems or hurdles (e.g., difficult navigation). Use bullet points with 1 sentence each. Be concise and avoid redundancies.  Describe any mentioned facilitators regarding use technology (e.g., higher education, higher digital literacy. Use bullet points with 1 sentence each. Be concise and avoid redundancies.  If the study includes patients and informal caregivers and barriers/facilitators differ among those groups, then report separately.  Format example:  Barriers:  - ....  - ....  Facilitators:  - .....  - ..... |
| Outcomes | There may be multiple outcomes or endpoints. If so, include all of them, as long as they are described outcomes or endpoints. Next to each outcome mention in brackets if it is related to patients, informal caregivers or care. Numerate and report as show in the example below:  1. blood glucose levels (patients)  2. anxiety (patients, informal caregivers)  3. burden (informal caregivers)  4. quality of care (care)  5. technology satisfaction (patients, informal caregivers)  Avoid mentioning any study findings here. Just mention the outcomes/endpoints.  If no outcomes were measured but just discussed/addressed (e.g., in the case of a qualitative study add a final sentence saying "outcomes not quantitatively measured"  Be as precise as possible. |
| Main findings | Summarize the results or conclusions of the study. Use numerated bullet points corresponding to the outcomes mentioned in column "Outcomes". Each bullet point should consist of only one concise sentence.  End with one sentence of main study takeaway. Make sure they convey the most important takeaways from the study. Avoid being redundant.  Format as shown below:  Outcomes  1.  2.  3.  ....  Main takeaway:  Here provide one sentence that summarizes the study findings. Only what is mentioned. Do not extrapolate. |
| PROs? | Does the study include/report patient reported outcomes? Answer with yes or no. If yes, briefly describe which. This are only outcomes reported by patients (NOT caregivers). If no, just say "No" |
