# Supplementary material for: Digital Informal Care: The Use of Technology in Family Care. A Scoping Review
Source: Public Health Rev. 2026 Jan 23;46:1608872. doi: 10.3389/phrs.2025.1608872 (PMC12876001; doi:10.3389/phrs.2025.1608872)
Supplement: Supplementary file 3 [file DataSheet1.docx]

**Supplementary File 1: Medline search strategy**

| **Database** | **Tailored search strategy and hits** | **Filters** |
| --- | --- | --- |
| **MEDLINE (via PubMed)** | (“famil* car*”[Title] OR “informal car*”[Title] OR “home car*”[Title] OR “parent* car*”[Title] OR “spous* car*”[Title]) AND (technolog*[Title] OR digital*[Title] OR “mobile health”[Title] OR m-health[Title] OR mhealth[Title] OR smartphone*[Title] OR wearable*[Title] OR smartwatch*[Title] OR e-health[Title] OR ehealth[Title] OR internet[Title] OR internet-based[Title] OR web-based[Title] OR computer-based[Title] OR online[Title] OR chatbot*[Title] OR virtual OR app[Title] OR application*[Title] OR telemed*[Title] OR telemedicine[Mesh] OR “artificial intelligence”[Title] OR “artificial intelligence” [Mesh]) NOT review[Title] | **Year:** 2019-2024  **Language:** English  **NOT:** reviews |
| **Web of Science** | TI=("family care*" OR "informal care*" OR “home car*” OR “parent* car*” OR “spous* car*”) AND TI=(technolog* OR digital* OR “mobile health” OR m-health OR mhealth OR smartphone* OR wearable* OR smartwatch* OR e-health OR ehealth OR internet OR internet-based OR web-based OR computer-based OR online OR chatbot* OR virtual OR app OR application* OR telemed* OR “artificial intelligence”) | **Year:** 2019-2024  **Language:** English  **NOT:** patent, awarded grant, letter, book, editorial material, case report, retraction, reviews, and news |
| **CINAHL** | ((TI “family car*” OR TI “informal car*” OR TI “home car*” OR TI “parent* car*” OR TI “spous* car*”) AND (TI technolog* OR TI digital* OR TI “mobile health” OR TI mhealth OR TI mhealth OR TI smartphone* OR TI wearable* OR TI smartwatch* OR TI e-health OR TI ehealth OR TI internet-based OR TI web-based OR TI computer-based OR TI chatbot* OR TI virtual OR TI app OR TI application* OR TI telemed* OR TI online OR TI internet)) | **Year:** 2019-2024  **Language:** English |
